# Inequities in quality perinatal care in the United States during pregnancy and birth after cesarean

**DOI:** 10.1371/journal.pone.0274790

**Published:** 2022-09-22

**Authors:** Bridget Basile Ibrahim, Saraswathi Vedam, Jessica Illuzzi, Melissa Cheyney, Holly Powell Kennedy

**Affiliations:** 1 Yale University School of Nursing, Orange, CT, United States of America; 2 Department of Family Practice, Birth Place Lab, Faculty of Medicine, University of British Columbia, Vancouver, BC, Canada; 3 Obstetrics, Gynecology & Reproductive Sciences, Yale School of Medicine, New Haven, CT, United States of America; 4 Anthropology Department, School of Language, Culture and Society, College of Liberal Arts, Oregon State University, Corvallis, OR, United States of America; 5 Department of Midwifery, Yale University School of Nursing, Orange, CT, United States of America; New York University Grossman School of Medicine, UNITED STATES

## Abstract

**Objective:**

High-quality, respectful maternity care has been identified as an important birth process and outcome. However, there are very few studies about experiences of care during a pregnancy and birth after a prior cesarean in the U.S. We describe quantitative findings related to quality of maternity care from a mixed methods study examining the experience of considering or seeking a vaginal birth after cesarean (VBAC) in the U.S.

**Methods:**

Individuals with a history of cesarean and recent (≤ 5 years) subsequent birth were recruited through social media groups to complete an online questionnaire that included sociodemographic information, birth history, and validated measures of respectful maternity care (Mothers on Respect Index; MORi) and autonomy in maternity care (Mother’s Autonomy in Decision Making Scale; MADM).

**Results:**

Participants (N = 1711) representing all 50 states completed the questionnaire; 87% planned a vaginal birth after cesarean. The most socially-disadvantaged participants (those less educated, living in a low-income household, with Medicaid insurance, and those participants who identified as a racial or ethnic minority) and participants who had an obstetrician as their primary provider, a male provider, and those who did not have a doula were significantly overrepresented in the group who reported lower quality maternity care. In regression analyses, individuals identified as Black, Indigenous, and People of Color (BIPOC) were less likely to experience autonomy and respect compared to white participants. Participants with a midwife provider were more than 3.5 times more likely to experience high quality maternity care compared to those with an obstetrician.

**Conclusion:**

Findings highlight inequities in the quality of maternal and newborn care received by birthing people with marginalized identities in the U.S. They also indicate the importance of increasing access to midwifery care as a strategy for reducing inequalities in care and associated poor outcomes.

## Introduction

Recognizing the need to humanize birth [1], the World Health Organization and leading maternity care scholars have incorporated respectful maternity care as a central tenet of high quality care, regardless of birth setting or technology and resources available in the country [2–5]. Person-centered maternity care is defined as “care that is respectful of and responsive to women’s preferences, needs, and values [and] is a core component of quality maternity care” [6]. For the purposes of this study, we chose to employ the term “higher quality maternity care” to describe perinatal care that is respectful and facilitates a level of autonomy preferred by the birthing person. In keeping with the self-identification of study participants, we employ the term “women” throughout this manuscript though we recognize that not all people who give birth identify as women and have varying gender identities and preferences for language.

Prior studies have found that women who have a hospital-based vaginal birth after cesarean (VBAC) [7] and those who decline care [8] are more likely to experience mistreatment during childbirth. Women who desired a VBAC, but were unable to plan one, were less likely to experience respectful maternity care or to have autonomy in their decision making for their maternity care than women who planned a VBAC [9]. Recent studies found that individuals who reported a difference of opinion with their providers about the right care for themselves or their baby were significantly more likely to report mistreatment or to feel disrespected and coerced by their provider [7, 9, 10].

Black and Indigenous women in the United States (U.S.) are significantly more likely to die within a year of giving birth [11, 12] and experience disproportionately higher rates of severe maternal morbidity [12, 13]. Nearly half of these maternal events [11] are preventable through improving quality of care [14]. Inequities in the quality of preconception, prenatal, intrapartum, and postpartum care may contribute to racial disparities in maternal health outcomes [11, 15, 16]. Notably, when mode of delivery is disaggregated by race, Black women in the U.S. have the highest rates of cesarean birth, despite similar predisposing factors [17–20].

Improving maternal health and health equity is a key priority of the U.S. Surgeon General [21], and the U.S. Department of Health and Human Services [22]. Racism and racial discrimination are linked to poor health [23, 24], and specifically, negative birth outcomes for women of color and their infants [24–27]. Mistreatment during pregnancy and childbirth has been associated with both short- and long-term adverse mental health outcomes that include pain and suffering, postpartum depression and post-traumatic stress disorder, fear of birth, negative body image, and feelings of dehumanization [28–30].

The purpose of this paper is to describe quantitative findings from a larger mixed methods study designed to investigate how U.S. women who were interested in considering a VBAC experienced maternity care in their subsequent pregnancy and childbirth. In this analysis, we examine associations between experiences of higher quality maternity care with sociodemographic characteristics that may impact the quality of maternity care, such as race, ethnicity, insurance status, model of care, and geographic region. Specifically, we explore the experiences of participants with intersecting identities, such as racialized identity and low socioeconomic status, that place individuals in disenfranchised (or excluded) social positions and at higher risk for poor birth outcomes.

## Materials and methods

In this paper, we report results from the survey portion of a convergent, parallel, mixed methods [31, 32] study describing women’s experiences of pregnancy and birth after cesarean in the United States. After Institutional Board Approval (IRB; Yale University IRB protocol # 2000021384), data were collected via a web-based questionnaire from May to October 2018. Signed informed consent was waived by the IRB. The questionnaire was preceded by an online written consent form to which participants had to indicate their agreement before beginning the survey.

### Sample and recruitment procedures

English-speaking adults who had experienced cesarean birth and had a subsequent child in the United States within the past 5 years were eligible to participate. Participants were included irrespective of the final mode of birth for their subsequent birth. The survey design and measures, data collection, sampling, recruitment, and results related to VBAC have been previously described in detail [9]. In brief, we designed, pilot-tested, revised, and then distributed a cross-sectional, retrospective, online questionnaire via the Qualtrics (Provo, UT) platform. Recruitment occurred through non-profit, peer-led, birth advocacy and support social media pages with more than 50,000 followers [33, 34].

### Study instruments

The questionnaire included a sociodemographic and birth history form and the Mothers on Respect Index (MORi) [35] and the Mother’s Autonomy in Decision Making scale (MADM) [36], both validated with U.S. populations. The MORi measures experiences of respectful maternity care [35] and the MADM measures agency in decision making during pregnancy, labor, and birth care [36]. Both instruments display high reliability and internal consistency [35, 36]. By completing the MADM scale, participants rate their ability to state their preferences in decision-making, whether different care options were presented, and if their choices were respected (7 items, scores range 7–42). On the MOR Index, participants describe their level of comfort with accepting or declining options for care, whether they felt poorly treated because of personal characteristics, and whether their treatment affected their willingness to ask questions (14 items, scores range 14–84). For both scales, respondents select one of 6 Likert-type response options to indicate agreement to statements. Higher scores on each scale indicate greater respect and autonomy when interacting with providers during pregnancy.

### Data management and analysis

Higher quality maternity care was defined based on the Quality Maternal and Newborn Care (QMNC) framework [5], and operationalized as measured by the MADM and MORi scales. In order to make the scores on the MADM and MORi more clinically relevant by capturing the overall experience of maternity care we created a dichotomous variable describing the quality of maternity care experienced. We created a dichotomous variable of lower versus higher quality maternity care wherein participants who scored in the lowest quartile of scores in our sample on the MORi (score < 57) and/or MADM (score < 23) were categorized as receiving lower quality maternity care. We categorized those who scored higher than the lowest quartile on both MADM and MORi scores in our sample as receiving higher quality perinatal care, as other studies using the MADM and MORi scales have done [37].

In order to explore associations with power and privilege between dominant versus non-dominant groups during interactions with healthcare providers, we assigned participants into dominant and non-dominant groups [38] and stratified our analyses based on the dominant and non-dominant identities of our participants to better highlight health inequities between groups [39]. A variable for BIPOC identification was created by dichotomizing those participants who self-identified as a race and ethnicity that has been historically or is currently marginalized within the United States (those participants who identified as any race or ethnicity other than white, non-Latinx) into Black, Indigenous, People of Color (BIPOC), and the remaining participants (those who self-identified as white, non-Latinx) into a non-BIPOC group. Participants who reported an annual household income of less than $50,000 were identified as low income, based on USDA criteria for a school age child in a family of four who would qualify for free and reduced school lunch with a 2017 annual income of $45,510 [40].

Descriptive statistics were computed and stratified by quality of maternity care experience and BIPOC identification. MADM and MORi median scores were calculated for the entire sample and stratified by BIPOC identification. Bivariate statistics were computed to determine if there were significant differences between sociodemographic characteristics and experiences of higher versus lower quality maternity care, as well as by BIPOC identification. Logistic regression was used to determine the likelihood of experiencing higher quality maternity care by various sociodemographic characteristics. Statistical analyses were completed using SAS Version 9.4 for Windows (Cary, NC). STROBE guidelines for reporting observational studies were used in reporting the findings of this study [41].

## Results

A total of 1711 participants, with a mean age of 34 years, completed the questionnaire (Table 1). Participants experienced a total of 4591 births; most experienced two (55%) or three (27%) births. The most commonly reported year of most recent birth was 2017. Most participants (87%) planned or attempted a VBAC, and 65% of participants had a VBAC for their first birth after cesarean. More than one quarter of participants (n = 487; 29%) reported one or more life adversities since becoming a parent. These included lacking health insurance, being unable to meet financial obligations, being unable to buy enough food, having their heat or electricity turned off, being unable to find work, housing instability, intimate partner violence, incarceration of self or partner, involvement of child protective services, and problems with drug/alcohol dependency. The full sample has been previously described in detail [9].

**Table 1:** Descriptive and bivariate statistics stratified by BIPOC identification for people in the United States with a recent birth after cesarean who completed an online self-administered questionnaire in 2018 (N = 1711).

Characteristic	n(%)	BIPOC ^a^	White, non-Latinx	Bivariate p-value
		n = 207 (12.1)	n = 1504 (87.9)	
**Highest level of education completed**				.08
No high school diploma	13 (0.8)	2 (1.0)	11 (0.7)	
High school diploma/GED	106 (6.2)	14 (6.8)	92 (6.1)	
Some college/2 year degree	515 (30.1)	79 (38.2)	436 (29.0)	
4 year degree	649 (37.9)	69 (33.3)	580 (38.6)	
Postgraduate degree	428 (25.1)	43 (20.8)	385 (25.6)	
**Annual household income**				.02
Less than $20,000	75 (4.4)	14 (6.8)	61 (4.1)	
$20,000-$50,000	474 (27.9)	70 (34.0)	404 (27.0)	
$50,000-$80,000	468 (27.5)	55 (26.7)	413 (27.6)	
$80,000-$125,000	441 (25.9)	38 (18.5)	403 (26.9)	
> $125,000	244 (14.3)	29 (14.1)	215 (14.4)	
**Low income^b^**	549 (32.1)	84 (40.6)	465 (30.9)	.005
**Insurance for prenatal care & 2^nd^ birth**				.001
Medicaid/government insurance	359 (21.0)	64 (30.9)	295 (19.6)	
Private	1270 (74.2)	136 (65.7)	1134 (75.4)	
Other	51 (3.0)	6 (2.9)	45 (3.0)	
No insurance	31 (1.8)	1 (0.5)	30 (2.0)	
**Region of residence ^c^**				< .0001
Northeast	194 (11.3)	24 (11.6)	174 (26.4)	
South	729 (42.6)	98 (47.3)	627 (41.7)	
Midwest	421 (24.6)	24 (11.6)	397 (26.4)	
West	367 (21.4)	61 (29.5)	306 (20.4)	
**Community of residence**				.37
Urban	375 (21.9)	52 (25.1)	323 (21.5)	
Suburban	1037 (60.6)	124 (59.9)	913 (60.7)	
Rural	299 (17.5)	31 (15.0)	268 (17.8)	
**Parity**				.40
2	950 (55.5)	114 (55.1)	836 (55.6)	
3	463 (27.1)	64 (30.9)	399 (26.5)	
4	182 (10.6)	21 (10.1.)	161 (10.7)	
5+	116 (6.8)	8 (3.9)	108 (7.2)	
**Provider for 1^st^ birth after cesarean**				.71
Midwife	569 (33.3)	63 (30.4)	506 (33.6)	
Family Doctor	40 (2.3)	4 (1.9)	36 (2.4)	
Obstetrician	1097 (64.1)	139 (67.2)	958 (63.7)	
No provider	5 (0.3)	1 (0.5)	4 (0.3)	
**Racial concordance with provider**	1386 (81.0)	18 (8.7)	1368 (84.0)	< .0001
**Travel time from home to location for second birth**				.53
< 30 minutes	1145 (67.0)	134 (65.1)	1011 (67.3)	
30–60 minutes	447 (26.2)	60 (29.1)	387 (25.8)	
> 60 minutes	116 (6.8)	12 (5.8)	104(6.9)	
**Doula for 2^nd^ birth**				.29
Yes	669 (39)	74 (35.7)	595 (39.6)	
No	1042 (61)	133 (64.3)	909 (60.4)	
**Plan/attempt VBAC**	1498 (87.6)	183 (88.4)	1315 (87.4)	.69
**Had a VBAC**	1107 (64.7)	128 (61.8)	979 (65.1)	.35
**Median Score Respect-MORi (IQR)**	71 (58–81)	70 (51–81)	71 (59–81)	.01
**Median Score Autonomy-MADM (IQR)**	34 (24–41)	33 (21–39)	34 (24–41)	.02

^a^ BIPOC = Black, Indigenous, People of Color; Participants identified race and Latinx ethnicity separately

^b^ Low income = annual household income of $50,000 or less

^c^ Regions of residence: Northeast (CT, MA, ME, NH, NJ, NY, PA, RI, VT), South (AL, AR, DC, DE, FL, GA, KY, LA, MD, MS, NC, OK, SC, TN, TX, VA, WV), Midwest (IA, IL, IN, KS, MI, MN, MO, ND, NE, OH, SD, WI), West (AK, AZ, CA, CO, HI, ID, MT, NM, NV, OR, UT, WA, WY).

Of the 1711 participants, 207 (12%) self-identified as a race or ethnicity other than white, non-Latinx and were categorized as BIPOC. Of the 549 (32%) low income participants, 84 (15%) were BIPOC, accounting for 41% of the BIPOC participants (Table 1). Medicaid was the payor for second births for 359 (21%) participants, 64 (18%) of whom were BIPOC, which accounts for 31% of the BIPOC participants. In bivariate analyses, BIPOC participants reported significantly different sociodemographic characteristics of lower household income, higher rates of Medicaid insurance, and higher rates of residence in the Southern United States.

Experiences of lower quality maternity care were reported by 534 (31%) of participants (Table 2). Characteristics indicating social disadvantage were overrepresented in the group who experienced lower quality maternity care: Black, Indigenous, Latinx, Asian and Multiracial groups (p = .004), participants with education less than a 4-year university degree (p < .0001), low income (p = .0002), and those with Medicaid insurance (p < .0001). Participants whose scores indicated lower quality maternity care were significantly more likely to report having an obstetrician (p < .0001), a male provider (p < .0001), shorter travel times for birth care (p = .003), and not having a doula present (p < .0001). Participants who did not plan (p < .0001) or obtain (p < .0001) a VBAC also reported experiencing lower quality maternity care significantly more often. BIPOC women also reported significantly lower median scores for autonomy (MADM) and respect (MORi) in their maternity care (Table 1).

**Table 2:** Quality of maternity care for people in the United States with a recent birth after cesarean (N = 1711).

Characteristic	n(%)	Lower quality maternity care ^a^	Higher quality maternity care ^a^	Bivariate
				p-value
		n = 534 (31.2)	n = 1177 (68.8)	
**Race**				.004
White	1568 (91.9)	472 (88.3)	1096 (93.1)	
Black	31 (1.9)	16 (3.0)	15 (1.3)	
American Indian/Alaska Native	18 (1.1)	4 (0.7)	14 (1.2)	
Asian	22 (1.3)	8 (1.5)	14 (1.2)	
Other	29 (1.7)	15 (2.8)	15 (1.3)	
Multiracial	38 (2.2)	18 (3.4)	20 (1.7)	
**Latinx ethnicity ^b^**				.07
Yes	105 (6.1)	41 (7.7)	64 (5.4)	
No	1604 (93.9)	492 (92.3)	1112 (94.6)	
**BIPOC identification ^c^**				.001
Yes	207(12.1)	84 (15.7)	123 (10.5)	
No	1504 (87.9)	450 (84.3)	1054 (89.5)	
**Highest level of education completed**				< .0001
No high school diploma	13 (0.8)	4 (0.7)	9 (0.8)	
High school diploma/GED	106 (6.2)	42 (7.9)	64 (5.4)	
Some college/2 year degree	515 (30.1)	206 (38.6)	309 (26.3)	
4 year degree	649 (37.9)	167 (31.3)	482 (41.0)	
Postgraduate degree	428 (25.1)	115 (21.5)	313 (26.6)	
**Annual household income**				.0003
Less than $20,000	75 (4.4)	37 (7.0)	38 (3.2)	
$20,000-$50,000	474 (27.9)	168 (31.7)	306 (26.0)	
$50,000-$80,000	468 (27.5)	139 (26.2)	329 (28.0)	
$80,000-$125,000	441 (25.9)	120 (22.6)	321 (27.3)	
> $125,000	244 (14.3)	66 (12.5)	178 (15.1)	
**Low income^d^**				.0002
Yes	549 (32.1)	205 (38.4)	344 (29.2)	
No	1162 (67.9)	329 (61.6)	833 (70.8)	
**Insurance for prenatal care & 2^nd^ birth**				< .0001
Medicaid/government insurance	359 (21.0)	146 (27.3)	213 (18.1)	
Private	1270 (74.2)	370 (69.3)	900 (76.5)	
Other	51 (3.0)	16 (3.0)	35 (3.0)	
No insurance/self-pay	31 (1.8)	2 (0.4)	29 (2.5)	
**Region of residence ^e^**				.06
Northeast	194 (11.3)	74 (37.4)	124 (62.6)	
South	729 (42.6)	229 (31.2)	499 (68.8)	
Midwest	421 (24.6)	114 (27.1)	307 (72.9)	
West	367 (21.4)	120 (32.7)	247 (67.3)	
**Community of residence**				.85
Urban	375 (21.9)	113 (21.2)	262 (22.3)	
Suburban	1037 (60.6)	325 (60.9)	712 (60.5)	
Rural	299 (17.5)	96 (18.0)	203 (17.2)	
**Parity**				< .0001
2	950 (55.5)	241 (45.1)	709 (60.2)	
3	463 (27.1)	167 (31.3)	296 (25.1)	
4	182 (10.6)	83 (15.5)	99 (8.4)	
5+	116 (6.8)	43 (8.0)	73 (6.2)	
**Provider for 1^st^ birth after cesarean**				< .0001
Midwife	569 (33.3)	89 (16.7)	480 (40.8)	
Family Doctor	40 (2.3)	11 (2.1)	29 (2.5)	
Obstetrician	1097 (64.1)	433 (81.0)	664 (56.4)	
No provider ^f^	5 (0.3)	-	-	
**Gender of provider for 2^nd^ birth**				< .0001
Female	1143 (66.8)	317 (59.4)	826 (70.2)	
Male	550 (32.1)	205 (38.4)	345 (29.3)	
*Missing*	*18 (1*.*1)*			
**Travel time from home to location for 2^nd^ birth**				.003
< 30 minutes	1145 (67.0)	368 (68.9)	777 (66.0)	
30–60 minutes	447 (26.2)	146 (27.3)	301 (25.6)	
> 60 minutes	116 (6.8)	20 (3.7)	96 (8.2)	
*Missing*	*3 (1*.*0)*			
**Doula for 2^nd^ birth**				< .0001
Yes	669 (39.0)	158 (29.6)	511 (43.4)	
No	1042 (61.0)	376 (70.4)	666 (56.6)	
**Plan/ attempt VBAC**				< .0001
Yes	1498 (87.6)	428 (80.1)	1070 (90.9)	
No	213 (12.4)	106 (19.9)	107 (9.1)	
**Had a VBAC**				< .0001
Yes	1107 (64.7)	259 (48.5)	848 (72.0)	
No	604 (35.3)	275 (51.5)	329 (28.0)	

^a^ Lower quality maternity care is defined as a score in the lowest quartile in the sample on the MADM and/or MORi (MADM < 23 and/or MORi < 57). Higher quality maternity care is defined as a score in the three highest quartiles on both the MADM (≥23) and the MORi (≥ 57).

^b^ Participants identified race and Latinx ethnicity separately

^c^ BIPOC = Black, Indigenous, Person of Color. Participant self-identifies as any race other than white or as Latinx ethnicity. (n = 207)

^d^ Low income = annual household income of $50,000 or less

^e^ Regions of residence: Northeast (CT, MA, ME, NH, NJ, NY, PA, RI, VT), South (AL, AR, DC, DE, FL, GA, KY, LA, MD, MS, NC, OK, SC, TN, TX, VA, WV), Midwest (IA, IL, IN, KS, MI, MN, MO, ND, NE, OH, SD, WI), West (AK, AZ, CA, CO, HI, ID, MT, NM, NV, OR, UT, WA, WY).

^f^ Participants who selected “No Provider” were excluded from analyses using the MADM and MORi scores as they did not have interactions with providers to describe in the instruments.

Participants reported varying levels of racial concordance with their maternity care provider for their first birth: American Indian Alaska Native (0%), Asian (4%), Black (21%), Multiracial (5%), Other (29%), White (84%). As a group, BIPOC participants reported racial concordance with their provider in less than 9% of cases, compared with 84% of white participants.

Rates of diagnoses of postpartum depression (PPD), posttraumatic stress disorder (PTSD) or birth trauma after their first birth also varied by participants’ race (Table 3). American Indian Alaska Native (PPD 16%, PTSD 11%) and Multiracial (PPD 20%, PTSD 15%) participants reported disproportionately high rates of perinatal mental health diagnoses.

**Table 3:** Postpartum mental health diagnoses after first birth by participant race, N = 1783.

	Participant Race (n)						
	Total	AI/AN	Asian	Black/AA	Multiracial	Other^a^	White
	1783	19	23	33	41	30	1637
**Diagnosis -%**							
Postpartum depression	16.2	15.8	8.7	0	19.5	10.3	16.7
PTSD/ birth trauma	9.5	10.5	4.3	3.0	14.6	10.3	9.5

AI/AN = American Indian/Alaska Native; AA = African American; PTSD = Posttraumatic stress disorder

^a “^Other” includes 1 Native Hawaiian and 20 who identified as Latinx for ethnicity and Other for race.

In logistic regression analyses, the likelihood of experiencing higher quality maternity care was significantly lower for BIPOC participants (OR 0.69, 95% CI 0.51, 0.95) compared with white participants, and for low-income BIPOC participants (OR 0.53, 95% CI 0.34, 0.82) compared with the remainder of the sample (Table 4). Those cared for by midwives were more than 3.5 times more likely to experience higher quality maternity care (OR 3.59, 95% CI 2.76, 4.68) relative to those who received care from an obstetrician.

**Table 4:** Crude odds ratios for the experience of higher quality maternity care for people with a history of cesarean as they experienced a subsequent pregnancy and birth in the United States, 2018, N = 1711.

Experienced higher quality maternity care ^a^	Odds Ratio	95% CI
Racial/ethnic identification		
BIPOC ^b^	0.69	0.51, 0.95
White, non-Latinx	reference	
Low income ^c^ BIPOC		
Yes	0.53	0.34, 0.82
No	reference	
Provider type		
Midwife	3.51	2.72, 4.54
Family physician	1.71	0.85, 3.47
Obstetrician	reference	

^a^ Higher quality maternity care is defined as a score in the three highest quartiles on the MADM (≥23) and on the MORi (≥ 57).

^b^ BIPOC = Black, Indigenous, Person of Color. Participant self-identifies as any race other than white or as Latinx ethnicity (n = 207).

^c^ Low income = annual household income of $50,000 or less; low income BIPOC (n = 84).

## Discussion

Key components of quality maternal and newborn care include respectful communication, care tailored to the birthing person’s circumstances and needs, and strengthens the person’s capabilities [5], which are aspects of care captured by the MADM and MORi scales [36]. In this national study of women with a history of cesarean, the most marginalized participants more frequently reported experiencing lower quality maternity care, according to the measures we employed. Black women, those with less education, those from a low-income household, and participants with Medicaid were overrepresented in the group that experienced lower quality maternity care during their experiences of pregnancy and birth after cesarean. BIPOC women were significantly less likely to experience higher quality maternity care and reported significantly lower scores on measures of autonomy and respectful maternity care, when compared with participants who self-identified with the dominant group of healthcare providers (white, non-Latinx).

We found that having a midwife as primary maternity care provider increased experiences of higher quality maternity care, and that autonomy and respect were more likely to be compromised for individuals who had an obstetrician for their primary maternity care provider, similar to the findings of the *Giving Voice to Mothers* study [7] and others [37, 42]. Midwifery models of care, which tend to be individualized, person-focused and collaborative, embody some of the core components of respectful, person-centered maternity care [43, 44]. Midwifery models of care are associated with lower rates of intervention, higher levels of respect, a focus on communication and information sharing [2, 5, 45] and high-quality care in support of normal, physiologic birth, including VBAC [5]. Further, midwifery care exemplifies the values described in the quality maternal and newborn care framework [5, 46] and has been shown to improve multiple outcomes for women and their newborns [47, 48].

Our findings are similar to other studies that have demonstrated loss of agency in decision making for childbirth [49, 50], and in particular, less agency in decision making and higher rates of mistreatment and discrimination reported by BIPOC and low-income participants [7, 37, 51, 52]. Lack of high-quality maternity care, exacerbated by poor communication and mistreatment during labor and birth can create a feeling of distrust toward caregivers [53] and distress in the maternity care provider-patient relationship [54].

Improving quality of maternity care, from preconception to postpartum, is critical to reducing health inequities [11, 55, 56]. Black and Indigenous women experience maternity care that is more discriminatory [51, 57], and less respectful and autonomous than white women [7]. Indigenous women are more likely to report experiences of mistreatment by maternity-care providers [7]. American Indian and First Nations communities have cited communication and institutional barriers, interpersonal problems, poverty, abuse, depression, substance use, and a lack of trust in Indian Health Service providers as affecting the quality of their prenatal care [58, 59]. Black women have similarly perceived their prenatal care to be of poorer quality [60]. BIPOC women’s experiences of poor communication from providers, coercion in their reproductive care, and discrimination increase levels of health care system distrust, contributing to and exacerbating racial disparities in health care [61]. These factors also contribute to lower utilization of preventative health services among lower-income Black Americans [62, 63].

BIPOC participants generally reported low rates of racial concordance with their maternity care providers. Saha and colleagues found that satisfaction is greater and people more frequently participate in preventative care when there is racial concordance between a person and their provider [64]. Unfortunately, achieving racial concordance with their maternity care provider may be challenging for BIPOC people as less than 6% of midwives identify as people of color [65] and a small portion of obstetricians are Black (11%) and Latinx (7%) [66]. When racial concordance is not possible, anti-racist and cultural humility trainings for providers and hospital staff [54, 67–69] may help to lessen the experiences of mistreatment due to racial discrimination among women of color.

Experiencing mistreatment, disrespectful care, and lack of autonomy, as well as the stress and anxiety of “fighting” for a VBAC [9] potentially has long lasting effects for both women and their children. Even mild stress in pregnancy can have negative influences on physiology and psychology for both mother and the developing child, and contribute to negative pregnancy outcomes such as preterm birth and fetal growth restriction [70]. Recent evidence from population level data links high rates of obstetric interventions like cesareans to postnatal maternal and neonatal morbidities and long term adverse child health consequences [71].

Participants who identified as American Indian and Multiracial reported disproportionately higher rates of postpartum depression and birth trauma after their first births. Notably, they also had very low rates of racial concordance with their maternity care providers. Higher rates of posttraumatic stress disorder symptoms were reported after unplanned cesarean [72]. Experiences, even those that may be considered routine by healthcare professionals can cause trauma during childbirth [73] and many women exhibit posttraumatic stress symptoms, while a smaller but still significant percentage meet diagnostic criteria for posttraumatic stress disorder due to their childbirth experiences [74]. These negative, fearful, or traumatic experiences may result in the release of catecholamines in early labor, causing labor to stall [75] and result in a “failure to progress” labor and subsequent cesarean. These experiences may also result in decreased trust in and utilization of the healthcare system in the future, which would lead to decreased screening and follow-up, possibly for the women and their children who need it the most.

### Strengths and limitations

A significant strength of this study is that it captured the experiences of a large and geographically diverse sample of women with a history of cesarean. However, our sample is not representative of the racial and ethnic diversity of U.S. individuals with a history of cesarean. [76]. Additionally, the majority (64.7%) of participants had a VBAC, well above the national VBAC rate of 13.9% [77]. Our findings are subject to self-selection bias due to recruitment in birth advocacy social media interest groups. Individuals who were active participants in social media birth advocacy were more likely to have been made aware of the opportunity to participate in this study. A further limitation is the sociodemographic homogeneity and of the sample despite significant efforts to reach out to birth justice groups for women of color, which limited adequate investigation of these potential interactions of intersecting identities. An additional limitation is our English-speaking inclusion criteria which limited our ability to reach non-English speaking people in the U.S. who may be further marginalized in the English-dominant U.S. health system. Further, due to the retrospective nature of the study, we were not able to measure the preferred amount of decision-making autonomy each participant desired during their pregnancy and birth.

### Implications for health equity

According to the World Health Organization, women’s experiences of care are equally important to the quality of clinical care provided [78, 79]. Respectful maternity care is an integral part of high quality care [44, 80] and human rights based respectful maternity care can improve women’s experiences and address health inequalities [4]. Our results indicate a disconnect between the aim of delivering person-centered, equitable care and our participants’ experiences of care.

This study illuminates the need for further research in many areas related to health equity in maternal health. In their recent integrative review, Sonderlund and colleagues found that experiences of discrimination predict a range of adverse birth outcomes and physiological markers of allostatic load in mother and child and may be contributing to health inequities [81]. Sudhinaraset and colleagues found that women who experienced more person-centered maternity care were less likely to report maternal or newborn complications or screen positive for depression [6]. Further research exploring the long-term and intergenerational biosocial, physiologic, and psychologic effects of quality of maternity care, especially focusing on marginalized groups (socioeconomically disadvantaged people, racial and ethnic minorities, and rural residents) is necessary.

Our study highlights the inequities in experiences of quality maternity care for a large cohort of women with a history of cesarean birth from all 50 U.S. states. Marginalized and socially disadvantaged women were the least likely to experience high-quality respectful, person-centered maternity care, even while they are the most at risk for adverse birth outcomes. Policy supports to improve access to midwifery care and doula services for Medicaid participants would increase access to experiences of respectful and high quality maternity care. Additional research is indicated to further quantify and measure quality maternity care [78] and to develop interventions that promote respectful maternity care, in both high and low resource settings [82].
